# Antitumor Activity of Noscapine in Combination with Doxorubicin in Triple Negative Breast Cancer

**DOI:** 10.1371/journal.pone.0017733

**Published:** 2011-03-15

**Authors:** Mahavir B. Chougule, Apurva R. Patel, Tanise Jackson, Mandip Singh

**Affiliations:** 1 College of Pharmacy, University of Hawaii, Hilo, Hawaii, United States of America; 2 College of Pharmacy, Florida A&M University, Tallahassee, Florida, United States of America; Florida International University, United States of America

## Abstract

**Background:**

The aim of this study was to investigate the anticancer activity and mechanism of action of Noscapine alone and in combination with Doxorubicin against triple negative breast cancer (TNBC).

**Methods:**

TNBC cells were pretreated with Noscapine or Doxorubicin or combination and combination index values were calculated using isobolographic method. Apoptosis was assessed by TUNEL staining. Female athymic Nu/nu mice were xenografted with MDA-MB-231 cells and the efficacy of Noscapine, Doxorubicin and combination was determined. Protein expression, immunohistochemical staining were evaluated in harvested tumor tissues.

**Results:**

Noscapine inhibited growth of MDA-MB-231 and MDA-MB-468 cells with the IC_50_ values of 36.16±3.76 and 42.7±4.3 µM respectively. The CI values (<0.59) were suggestive of strong synergistic interaction between Noscapine and Doxorubicin and combination treatment showed significant increase in apoptotic cells. Noscapine showed dose dependent reduction in the tumor volumes at a dose of 150–550 mg/kg/day compared to controls. Noscapine (300 mg/kg), Doxorubicin (1.5 mg/kg) and combination treatment reduced tumor volume by 39.4±5.8, 34.2±5.7 and 82.9±4.5 percent respectively and showed decreased expression of NF-KB pathway proteins, VEGF, cell survival, and increased expression of apoptotic and growth inhibitory proteins compared to single-agent treatment and control groups.

**Conclusions:**

Noscapine potentiated the anticancer activity of Doxorubicin in a synergistic manner against TNBC tumors via inactivation of NF-KB and anti-angiogenic pathways while stimulating apoptosis. These findings suggest potential benefit for use of oral Noscapine and Doxorubicin combination therapy for treatment of more aggressive TNBC.

## Introduction

Approximately 30–40% of breast cancers are estrogen receptor (ER) negative and the triple negative breast cancer (TNBC i.e. negative for ERα, PR and Her2 amplification) are the most clinically aggressive breast tumors [1], [2]. TNBC relapses quickly in response to clinical treatment as this subtype of breast cancer has a high histological grade and poor prognosis [3]. Patients with TNBC, which account for about 10–17% of all breast cancer cases [4], are often unresponsive to endocrine agents such as tamoxifen and less responsive to standard adjuvant therapy [5]. Specific targeted therapies are not available to improve clinical outcome among TNBC patients. [4], [5] TNBC do not respond to endocrine agents or trastuzumab and can only be treated with chemotherapy and treatment options for these tumors are limited by frequent de novo or acquired resistance to chemotherapy [6]. The limited availability of current systemic treatment options for TNBC necessitates the search for newer chemotherapeutic regimens. A promising target for the treatment of these ER^−^ breast tumors is the microtubule cytoskeleton [7]. The effectiveness of microtubule-interfering agents, taxanes and vinca alkaloids in treatment of various cancers has been well studied [8]. However, the clinical utility of taxanes has been limited due to drug-resistance, need of i.v. infusion over a long period of time and associated toxicities [9], [10]. This has prompted search for microtubule targeting agent that may be administered orally, display favorable toxicity profiles and have better therapeutic indices in the treatment of TNBC. Noscapine attenuates microtubule dynamics just enough to activate the mitotic checkpoints to stop cell cycle and does not alter the steady state monomer/polymer ratio of tubulin [11], [12]. Noscapine showed antitumor activity against a variety of cancer types (melanoma [13], ovarian [14], lymphoma [15], human myelogenous leukemia [16], gliobastoma [17], lung, [18] and breast [19]) both in vitro and in vivo while exerting minimal adverse side effects. Furthermore, Noscapine also showed little or no toxicity to the kidney, heart, liver, bone marrow, spleen, or small intestine and did not inhibit primary humoral immune responses in mice. Previous studies demonstrated that oral administration of Noscapine at 120 mg/kg and 300 mg/kg showed significant reduction in tumor volume in MCF-7 [19] and MDA-MB-231 [20] xenografts in nude mice. However, the effectiveness of Noscapine in combination with other anticancer agents for treatment of TNBC has not been studied yet. At present, the lack of highly effective therapeutic targets for TNBC leaves standard chemotherapy, for example use of combination of anthracycline and taxane, however these agents are insufficiently efficacious [21]. Doxorubicin is an anthracycline drug which is used as a chemotherapeutic agent for patients with metastatic breast cancer and has shown overall response rates between 35 and 50% in patients with TNBC who have not previously received chemotherapy [22]. Despite its excellent anti-tumor activity, Doxorubicin has a relatively low therapeutic index and its clinical utility is limited due to acute and chronic toxicities such as myelosuppression, immunosupression and dose-cumulative cardiotoxicity [23]. Therefore, combination treatment with another highly effective novel non-toxic drug which can lower the dose of chemotherapeutic agents would be desirable.

Given the challenge in treating ER^−^ breast tumors and its inherent poor prognosis, the use of Noscapine in combination Doxorubicin will have major clinical implications for the treatment of ER^−^ breast cancer. Based on the individual activity of these agents and their distinct mechanisms of action, we hypothesize that Noscapine in combination with Doxorubicin will produce additive or synergistic cytotoxic effects in human TNBC *in vitro* and *in vivo* possibly by inactivation of NF-KB and also via antiangiogenic and apoptotic activity. The objectives of this study are (a) to examine the anticancer activity of Noscapine alone and combination with Doxorubicin against TNBC cells, and (b) evaluate the antitumor effect of Noscapine alone and combination in mice bearing MDA-MB-231 xenograft TNBC tumors and elucidates underlying mechanism of action.

## Materials and Methods

Noscapine and Doxorubicin were purchased from Sigma Chemicals, St. Louis, MO, USA and Spectrum Chemicals USA. The human breast cancer cell lines MDA-MB-231 and MDA-MB-468 were obtained from American Type Culture Collection (Rockville, MD, USA). Cells were grown in DMEM:F12K medium (Sigma, St. Louis, MO, USA) supplemented with 10% fetal bovine serum. The cell culture media contained antibiotic antimycotic solution of penicillin (5,000 U/ml), streptomycin (0.1 mg/ml), and neomycin (0.2 mg/ml). The cells were maintained at 37°C in the presence of 5% CO_2_ in air. The cells were maintained at 37°C in the presence of 5% CO_2_. All other chemicals were either reagent or tissue culture grade.

### Animals

Female Nu/Nu mice (six weeks old form Harlan, Indianapolis, IN) were grouped and housed (n = 8 per cage) in sterile microisolator caging unit supplied with autoclaved Tek-Fresh bedding. The animals were kept under controlled conditions of 12∶12 hour light: dark cycle, 22±2°C and 50±15 percent relative humidity. The mice were fed (irradiated rodent chow Harlan Teklad) and autoclaved water ad libitum. The animals were housed at Florida A and M University in accordance with the standards of *the Guide for the Care and Use of Laboratory Animals* and the Association for Assessment and Accreditation of Laboratory Animal Care.

### *In-vitro* cytotoxicity studies

The MDA-MB-231 or MDA-MB-468 TNBC cell lines were plated in 96-well micro titer plates, at a density of 1×10^4^ cells/well and allowed to incubate overnight and were treated with various dilutions of Noscapine made in cell growth medium (10 to 160 µM) from Noscapine stock solution in DMSO. The cells were incubated for 72 h at 37±0.2°C in a 5% CO_2_-jacketed incubator. To study the interaction between Noscapine and Doxorubicin, the MDA-MB-231 or MDA-MB-468 cells were treated with various dilutions of Doxorubicin in the presence or absence of Noscapine at 10, 20 and 30 µM. The plates were incubated for 72 h at 37±0.2°C in a 5 percent CO_2_-jacketed incubator. Cell viability in each treatment group was determined by crystal violet dye assay. Separate study was done to find out the IC_50_ values of Noscapine and Doxorubicin for the MDA-MB-231 and MDA-MB-468 cells.

### Data analysis for the combination treatments

The percentage of cell survival as a function of drug concentration was then plotted to determine the IC_50_ value (the drug concentration needed to prevent cell proliferation by 50%) [18], [24]. The interactions between Doxorubicin and Noscapine were evaluated by isobolographic analysis, a dose-oriented geometric method of assessing drug interactions [25]. For 50 percent toxicity, the combination index (CI) values were calculated based on the equation stated below.
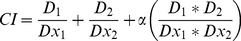
(1)Where,

Dx1 = Dose of drug 1 to produce 50 percent cell kill alone;
D1 = Dose of drug 1 to produce 50 percent cell kill in combination with D2;
Dx2 = Dose of drug 2 to produce 50 percent cell kill alone;
D2 = Dose of drug 2 to produce 50 percent cell kill in combination with D1;
α = 0 for mutually exclusive or 1 for mutually non-exclusive modes of drug action.

### Induction of apoptosis in MDA-MB-231 cells

To detect apoptotic cells, the ApoTag Red *In Situ* Apoptosis detection kit ^R^ (Chemicon ^R^ International, CA, USA) was used. MDA-MB-231 cells were plated at a density of 1×10^6^ cells/well in 6-well plates and incubated overnight. Cells were treated with Doxorubicin (0.4 µg/ml), or Noscapine (30 µM), or combination. Untreated cells were used as control. After 72 h, cells were fixed in 4% paraformaldehyde and mounted onto slides using Cytospin ^R^ (Shandon). Equilibration buffer was added to slides and incubated for 10 minutes followed by incubation in working strength TdT enzyme at 37°C for 1 hour. The slides were incubated in stop/wash buffer for 10 minutes at room temperature. Working strength anti-digoxinenin conjugate (rhodamine) was added to each slide for 30-minute incubation at room temperature. The images on the slides were visualized with an Olympus BX40 fluorescent microscope equipped with a computer-controlled digital camera (DP71, Olympus Center Valley, PA, USA). To quantify the apoptotic cells from terminal deoxynucleotidyl transferase-mediated nick end labeling (TUNEL) assay, 100 cells from 6 random microscopic fields were counted.

### *In-vivo* antitumor effect against MDA-MB-231 tumors

The adherent MDA-MB-231 tumor cells were washed with PBS, harvested from confluent cultures by 5-minute exposure to 0.25 percent trypsin and 0.02 percent EDTA solution in an incubator. Trypsinization was stopped with medium containing 10 percent FBS. The cells were centrifuged at 500 g for 4 min at 4°C and the floating cells in the supernatant were discarded. The cell pellet was resuspended in medium containing 10 percent FBS and mixed thoroughly. Trypan blue staining was used to determine the number of viable cells. The resuspended cells were dilutions of 3×10^6^ cells/100 µl were prepared in cell growth medium. The 100 µl of cell suspension was injected subcutaneously into right flank area of each mouse [18]. The protocol for *in-vivo* experiments with nude mice was approved by the Animal Care and Use Committee, Florida A and M University, Tallahassee, FL. The mice were randomized into vehicle control and treatment groups (n = 8) when xenografts were palpable with a tumor size of approximately 50 mm^3^. The mice were treated with i) 160 µl of vehicle; ii) Noscapine (150 mg/kg/day); iii) Noscapine (300 mg/kg/day); iv) Noscapine (450 mg/kg/day); v) Noscapine (550 mg/kg/day); vi) Doxorubicin (1.5 mg/kg/week, i.v.), vii) Noscapine (300 mg/kg/day)+Doxorubicin (1.5 mg/kg/week. i.v.). To check for evidence of toxicity, the animals were weighed twice weekly. The tumor dimensions were measured using a linear caliper and tumor volume was calculated using following equation:
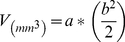
(2)Where,

V = tumor volume
a = largest diameter of tumor
b = smallest diameter of tumor

The mice were fed with food and water ad libitum. On day 38, all animals were sacrificed by exposure to a lethal dose of halothane in a desiccator. After dissection and removal of the tumor tissues, the tumors were washed in sterile PBS. For immunohistochemistry (IHC), and TUNEL assay procedures, some of the tumors were fixed in formalin while others were rapidly frozen in liquid nitrogen and stored in −80°C.

### Western blotting analysis of xenograft MDA-MB-231tumors

Tumor tissues harvested at 38 days post tumor implantation from control, Noscapine, Doxorubicin and combination treated mice were cut into small pieces and homogenized in PBS. The homogenate was centrifuged at top speed for 10 min to sediment the tissue fragments. The proteins were extracted using RIPA buffer (50 mM Tris-HCL, pH 8.0, with 150 mM sodium chloride, 1.0 percent Igepal CA-630 (NP-40), 0.5 percent sodium deoxychlolate, and 0.1 percent sodium dodecyl sulfate) with protease inhibitor (500 mM phenylmethylsulfonyl fluoride). Samples were vortexed, incubated on ice for 30 min, centrifuged again and the supernatants were stored at −80°C. For WB, equal amounts of supernatant protein (50 µg) from the control and different treatments were denatured by boiling for 5 min in SDS sample buffer (0.25 M Tris-HCl pH 6.8, 8% SDS, 30% Glycerol, 0.02% Bromophenol Blue and 10% 2-beta-mercaptoethanol), separated by 15% SDS-PAGE and transferred to nitrocellulose membranes for immunoblotting. The membranes were blocked with 5 percent skim milk in Tris-buffered saline with Tween 20 [10 mM Tris-HCl (pH 7.6), 150 mM NaCl, 0.5 percent Tween 20] and probed with NF-kβ (1∶500), IKBα (1∶500), P-IKBα (1∶500), Bax (1∶1000), Bcl_2_ (1∶1000), caspase 3 (1∶1000), cleaved caspase 3 (1∶1000), caspase 8 (1∶1000), caspase 9 (1∶1000), VEGF (1∶500), survivin (1∶500) and β-actin antibodies (1∶500). All primary antibodies were purchased from Cell Signaling Technology (Beverly, MA). Bound antibodies were revealed with HRP conjugated secondary antibodies (1∶2000) using SuperSignal West pico chemiluminescent solution (Pierce, Rockford, IL). Beta actin (Santa Cruz Biotechnology) protein was used as a loading control. The densitometric analysis of the bands was performed using the program ImageJ v1.33u.

### TUNEL assay of xenograft MDA-MB-231 tumors

Formalin-fixed tumor tissues harvested 38 days after tumor implantation were embedded in paraffin and sectioned (4–5 µm thick). DeadEnd™ Colorimetric Apoptosis Detection System (Promega, Madison, WI) was used to detect apoptosis in the tumor sections placed on slides according to the manufacturer's protocol. Briefly, the equilibration buffer was added to slides and incubated for 10 minutes followed by 10-minute incubation in 20 µg/ml proteinase K solution. The sections were washed in PBS and incubated with TdT enzyme at 37°C for 1 hour in a humidified chamber for incorporation of biotinylated nucleotides at the 3′- OH ends of DNA. The slides were incubated in horseradish peroxidase-labeled streptavidin to bind the biotinylated nucleotides followed by detection with stable chromagen DAB. The images on the slides were visualized with an Olympus BX40 light microscope equipped with a computer-controlled digital camera (DP71, Olympus Center Valley, PA, USA). Three slides per group were stained and apoptotic cells were identified by dark brown cytoplasmic staining.

### Immunohistochemistry for Cleaved Caspase 3 and VEGF Expression of MDA-MB-231 tumors

Tumor tissue sections prepared from formalin-fixed, paraffin-embedded tumor tissues were used for IHC studies according to the protocol specified in the SignalStain™ Cleaved Caspase-3 (Asp 175) IHC kit (Cell Signaling, Beverly, MA). The section slides were washed in xylene and hydrated in different concentrations of alcohol. The slides were heated in sodium acetate solution at 95°C for 10 minutes for antigen retrieval. The slides were washed three times in PBS and incubated with the primary antibody against cleaved caspase-3 overnight at 4°C. Horseradish peroxidase-conjugated secondary antibody was applied to locate the primary antibody. The specimens were stained with Nova Red stain and counterstained with hematoxylin. The presence of brown staining was considered a positive identification for activated caspase-3. For VEGF staining, the tissue sections were washed, hydrated and processed for antigen retrieval as described above for cleaved caspase-3 staining. The samples were incubated overnight at 4°C with either 1∶50 dilution of VEGF antibody incubated with biotinylated secondary antibody followed by streptavidin. The color was developed by exposing the peroxidase to a substrate-chromagen, which forms a brown reaction product. VEGF expression was identified by the brown cytoplasmic staining. The Olympus BX40 light microscope equipped with computer-controlled digital camera (DP71, Olympus Center Valley, PA, USA) was used to visualize the images on the slides.

### CD31 expression and Assessment of Microvessel Density of MDA-MB-231 tumors

Paraffin-embedded tumor tissues were deparaffinized and blocked for peroxidase activity as described under methodology for IHC for VEGF Expression. After washing with PBS, the sections were pretreated in citrate buffer in a microwave oven for 20 min at 92–98°C. After two washes with PBS, specimens were incubated in 10 percent normal goat serum (Atlanta Biologicals, GA,USA) for 20 min to reduce the nonspecific antibody binding. Subsequently, the sections were then incubated with a 1∶500 diluted mouse CD31 monoclonal antibody (Cell Signaling Tech, MA), which is recognized as an endothelial cell surface marker, at room temperature for 1 h, followed by a 30 min treatment with HRP Rabbit/Mouse (Santa Cruz Biotechnology, Santa Cruz, CA,USA). After three washes with PBS, the section was developed with diaminobenzidene-hydrogen peroxidase substrate, and lightly counterstained with hematoxylin. To calculate microvessel density (MVD), three most vascularised areas of the tumour (‘hot spots’) were selected and mean values obtained by counting vessels. A single microvessel was defined as a discrete cluster of cells positive for CD31 staining, with no requirement for the presence of a lumen. Microvessel counts were performed at ×400 (×40 objective lens and ×10 ocular lens; 0.74 mm^2^ per field).

### Statistics

One-way ANOVA followed by Tukey's Multiple Comparison Test was performed to determine the significance of differences among groups using GraphPad PRISM version 3.0 software (SanDiego, CA). Differences were considered significant in all experiments at *P*<0.01 (*, significantly different from untreated controls; ^**^, significantly different from Noscapine and Doxorubicin single treatments.

## Results

### Cell proliferation inhibition by Noscapine and synergistic behavior in combination with Doxorubicin

Noscapine inhibited proliferation of MDA-MB-231 and MDA-MB-468 cells with an IC_50_ value of 36.16±3.76 and 42.7±4.3 µM respectively. Doxorubicin showed IC_50_ of 0.21±0.09 µg/ml and 0.20±0.08 µg/ml against MDA-MB-231 and MDA-MB-468 TNBC cells respectively. The combined effects of Doxorubicin and Noscapine on cell proliferation were evaluated by isobolographic analysis method. The CI values ranged from 0.51±0.03 to 0.60±0.05 for 50 percent cell kill suggesting synergistic behavior between Noscapine and Doxorubicin against both TNBC cells (Table 1).

**Table 1:** Combination Index (CI) values of the interaction between Nos with Dox against human MDA-MB-231 and MDA-MB-468 TNBC cells.

Drug Combinations	MDA-MB-231		MDA-MB-468		
	CI at IC_50_	Interpretation	Drug Combinations	CI at IC_50_	Interpretation
Dox+Nos 10	0.55±0.03	Synergism	Dox+Nos 10	0.51±0.03	Synergism
Dox+Nos 20	0.59±0.04	Synergism	Dox+Nos 20	0.53±0.07	Synergism
Dox+Nos 30	0.60±0.05	Synergism	Dox+Nos 30	0.60±0.04	Synergism

The human lung cancer cell lines MDA-MB-231 and MDA-MB-468 breast cancer cells were obtained from American Type Culture Collection (Rockville, MD). Different concentrations of Nos were employed to study the effect on IC50 of Dox. Variable ratios of drug concentrations and mutually non-exclusive equations were used to determine the CI. The CI values represent mean of four experiments. CI>1.3: antagonism; CI 1.1–1.3: moderate antagonism; CI 0.9–1.1: additive effect; CI 0.8–0.9: slight synergism; CI 0.6–0.8: moderate synergism; CI 0.4–0.6: synergism; CI 0.2–0.4: strong synergism.

### Induction of apoptotic DNA fragmentation in MDA-MB-231 cells

**Figure 1:**
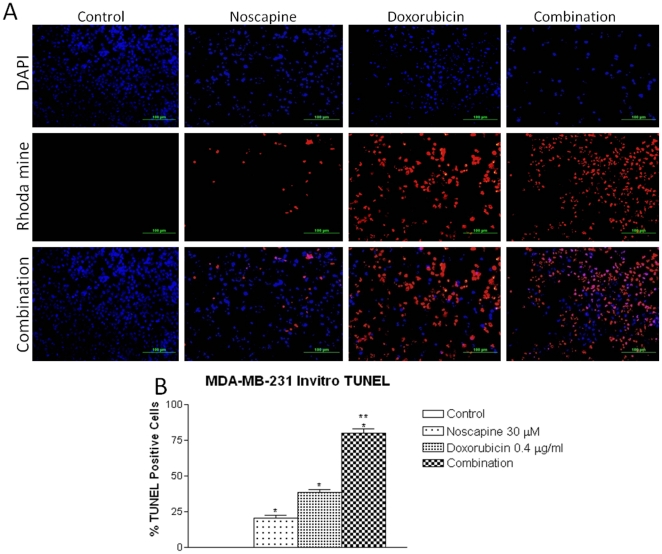
Fluorescence Micrographs of cells stained with rhodamine and DAPI after 72 h (A) with Doxorubicin 0.4 µg/ml , Noscapine 30 µM, and, Noscapine and Doxorubicin combination in MDA-MB-231 cells and (B) Quantitation of apoptotic MDA-MB-231 cells from TUNEL assay. DNA fragmentation indicated by positive staining (red) and nuclear condensation indicated by DAPI nuclear staining (blue). Micron bar = 100 µm. Cells were quantitated by counting 100 cells from 6 random microscopic fields. Data are expressed as mean+SD (N = 6). One-way ANOVA followed by post Tukey test was used for statistical analysis to compare control and treated groups. * *P*<0.01; all treatments significantly different from control and ** *P*<0.01; significantly different from Noscapine and Doxorubicin single treatments.

Figure 1 shows that apoptosis is induced in MDA-MB-231 cells following treatment with Doxorubicin, or Noscapine, or combination. Fig. 1A & 1B shows MDA-MB-231 cells undergoing apoptosis following treatment with Noscapine 30 µM compared to untreated cells. Combination treatment led to apoptosis in 79±4 percent of treated MDA-MB-231 cells compared to 32±3.0 percent and 22±2.0 percent in Doxorubicin and Noscapine respectively after 72 h (Fig. 1A and 1B). All treatments were significantly different from control (* *P*<0.01). Doxorubicin or Noscapine treatment was significantly different from combination treatment (**, *P*<0.001).

### Anti-tumor effect of Noscapine against MDA-MB-231 xenograft model

**Figure 2:**
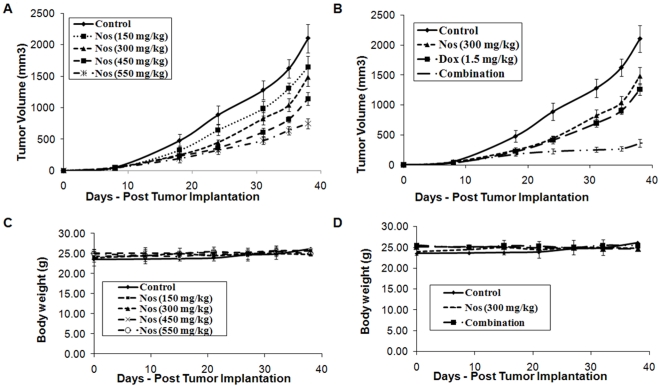
Progression profile of tumor growth kinetics of in-vivo antitumor effect of different doses of Noscapine alone (A) and in combination with Doxorubicin (B) on human MDA-MB-231 tumor xenograft model (tumor volumes, mm^3^ ± SEM), and measurement of body weight following Noscapine alone (C) and combination with Doxorubicin (D). Female nude mice with xenograft MDA-MB-231 tumor tumors received various treatments for 38 days starting on day 7 post tumor implantation. The mice were treated with Noscapine (150–550 mg/kg/day), Doxorubicin 1.5 mg/kg i.v. bolus, q3d×7 schedule, and Noscapine 300 mg/kg/day+Doxorubicin 1.5 mg/kg i.v. bolus, q3d×7 schedule. Control group received vehicle only. Statistical significance of the difference in tumor volume of treatment groups compared with control. *P*<0.01 (*, significantly different from untreated controls; ^**^, significantly different from Noscapine and Doxorubicin single treatments). Data presented are means and SE (n = 8). This experiment was repeated twice.

Figures 2A shows the tumor volume-time data profiles following Noscapine administration at a dose of 150–550 mg/kg/day by gastric lavage in mice xenografted with MDA-MB-231 tumors compared to control. At 38 days post tumor implantation, the tumor volumes were found to be 2102±225 mm^3^, 1648±172 mm^3^, 1485.93±146 mm^3^, 1139±103 mm^3^ and 748±83 mm^3^ (expressed as mean±SEM) in control, Noscapine 150, 300, 450 and 550 mg/kg/day treated mice, respectively (Fig. 2A). Oral administration of Noscapine at 150–550 mg/kg/day showed significant (p<0.01) reduction in tumor volume in MDA-MB-231 xenografts. However, Noscapine administered at 450 and 550 mg/kg/day showed very significant (p<0.001) reduction in tumor volume. At the end of the study period (38 days), there were 21, 29, 45, and 64 percent reduction in the tumor volume following Noscapine 150, 300, 450, 550 mg/kg/day treated mice respectively compared to control. We found that Noscapine treatment did not cause any apparent body weight loss in mice (Fig. 2C).

### Anti-tumor effect of Noscapine (300 mg/kg/day) and Doxorubicin combination in MDA-MB-231 xenograft model

The results (Fig. 2B) show that tumor volume significantly decreased after treatment with Doxorubicin (1.5 mg/kg/week i.v. bolus, *P*<0.01), Noscapine (300 mg/kg oral, *P*<0.01), or combination (P<0.001) compared to control. Tumor volume for the combination treatment averaged 361±64 mm^3^ compared with 1648±172 mm^3^ for Noscapine treatment or 1259±99 mm^3^ for Doxorubicin treatment on day 38 post tumor implantation. It is evident that combination treatment was most effective in inhibiting tumor growth compared to Doxorubicin or Noscapine treatments. Furthermore, we did not observe any weight loss or other signs of toxicity in the mice treated with combination or Noscapine or Doxorubicin (Fig. 2D).

### Inactivation of NF-KB, activation of proapoptotic and inhibition of antiapopototic proteins in MDA-MB-231 xenograft model

**Figure 3:**
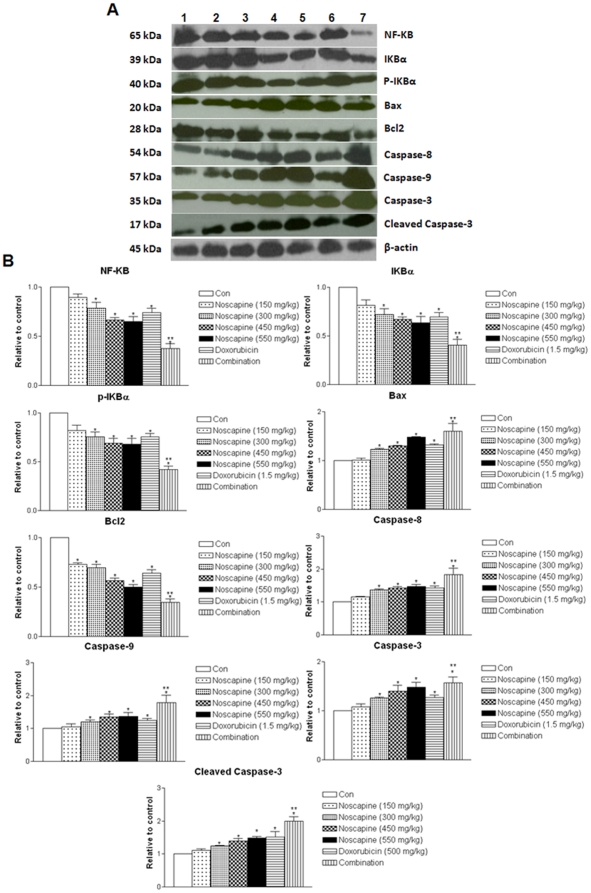
Western blotting of tumor tissue lysates to determine expressions apoptosis-related proteins (A) expression of NF-kβ, IKBα, P-IKBα, Bax, Bcl2, caspase 3, cleaved caspase 3, activated caspase 8 and activated caspase 9 proteins in tumor lysates by western blotting and (B) quantitation of apoptotic protein expression. Tumor tissue lysates harvested tumor tissues from control-untreated and treated groups were analyzed by western blotting for protein expressions. Lane 1 = control; Lane 2 = Noscapine 150 mg/kg/day; Lane 3 = Noscapine 300 mg/kg/day; Lane 4 = Noscapine 450 mg/kg/day; Lane 5 = Noscapine 550 mg/kg/day; Lane 6 = Doxorubicin 1.5 mg/kg i.v. bolus, q3d×7 schedule; Lane 7 = Combination (Noscapine 300 mg/kg/day+Doxorubicin1.5 mg/kg i.v. bolus, q3d×7 schedule). Similar results were observed in triplicate experiments. Protein expression levels (relative to β-actin) were determined. Mean ± SE for three replicate determinations. One-way ANOVA followed by post Tukey test was used for statistical analysis. *P*<0.01 (*, significantly different from untreated controls; ^**^, significantly different from Noscapine and Doxorubicin single treatments).

Noscapine treatments significantly (P<0.001) decreased expression of NF-kB, IkBα, P-IkBα, BCl_2_ and increased expression of cleaved PARP, Bax, activated caspase8, activated caspase 9, caspase 3 and cleaved caspase 3 proteins compared to control (Fig. 3) except Noscapine administered at 150 mg/kg/day (P>0.05). The observed protein responses were found to be dose dependant. The Bax/Bcl_2_ ratio of 0.62 was observed in control tumors, while the Bax/Bcl_2_ ratio of 0.8, 1.1, 1.3, and 1.6 were observed with Noscapine 150 mg/kg 300, 450, and 550 mg/kg treated tumors respectively. A non-significant (*P*>0.05) increase in Bax/Bcl_2_ ratio was observed with Noscapine 150 mg/kg/day, while a significant (*P*<0.001) increase in Bax/Bcl_2_ ratio was found with Noscapine 300, 450 and 550 mg/kg/day (Fig. 3A & 3B). Results illustrated in Fig. 3A and 3B show that Noscapine, Doxorubicin and combination treatment showed significant (*, p<0.001) increased expression of Bax, activated caspase8, activated caspase 9, caspase 3 and cleaved caspase 3 proteins and decreased expression of NF-KB, IKBα, P-IkBα and Bcl_2_ compared to control group. The expression of apoptotic and antiapoptotic proteins in combination treatment was significantly (*, **, p<0.01) different from single agent treatment groups.

### Effects on angiogenic and cell survival proteins in MDA-MB-231 xenograft model

**Figure 4:**
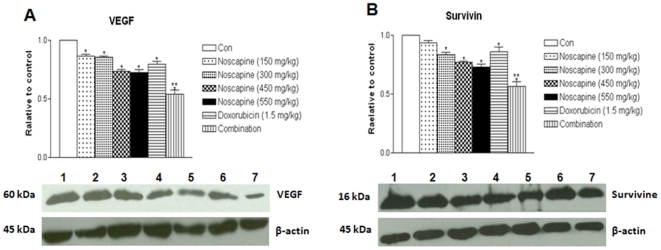
Western blotting of tumor tissue lysates to determine expressions angiogenesis-related proteins expression of (A) VEGF and (B) survivin proteins in tumors and quantitation of protein expression. Whole-cell lysates from control-untreated and treated tumors were analyzed by western blotting for protein expressions. Lane 1 = control; Lane 2 = Noscapine 150 mg/kg/day ; Lane 3 = Noscapine 300 mg/kg/day ; Lane 4 = Noscapine 450 mg/kg/day; Lane 5 = Noscapine 550 mg/kg/day; Lane 6 = Doxorubicin 1.5 mg/kg i.v. bolus, q3d×7 schedule; Lane 7 = Combination (Noscapine 300 mg/kg/day+Doxorubicin ). Similar results were observed in replicate experiments. Protein expression levels (relative to β-actin) were determined. Mean ± SE for three replicate determinations. One-way ANOVA followed by post Tukey test was used for statistical analysis. *P*<0.01 (*, significantly different from untreated controls; ^**^, significantly different from Noscapine and Doxorubicin single treatments).

We compared expression of angiogenic and survival protein in tumor lysates from control and treated mice by western blotting analysis using β-actin as loading control (Fig. 4). Noscapine treatment significantly (P<0.001) decreased expression of VEGF (Fig. 4A), and survivin (Fig. 4B) proteins except Noscapine at a dose of 150 mg/kg (P>0.05) compared to control. Noscapine treatment at 300, 450 and 550 mg/kg/day showed 0.14, 0.26 and 0.28 Fold decreased in VEGF expression inregressed tumor compared to vehicle treated control group. Combination treatment decreased expression of VEGF protein expression significantly (**, P<0.001) to 0.45-fold compared to 0.14-fold with Noscapine (*, P<0.01) and 0.20-fold with Doxorubicin (*, P<0.01) treatment, respectively of controls in regressed tumors (Fig. 4A). The expression of survivin protein were significantly decreased by 0.44 fold (*, P<0.01), 0.15 fold (*, P<0.05) and 0.08 fold (*, P<0.05) with combination, Doxorubicin and Noscapine treatment compared to control group respectively (Fig. 4B).

### DNA fragmentation and expression of cleaved caspase-3 in MDA-MB-231 xenograft model

**Figure 5:**
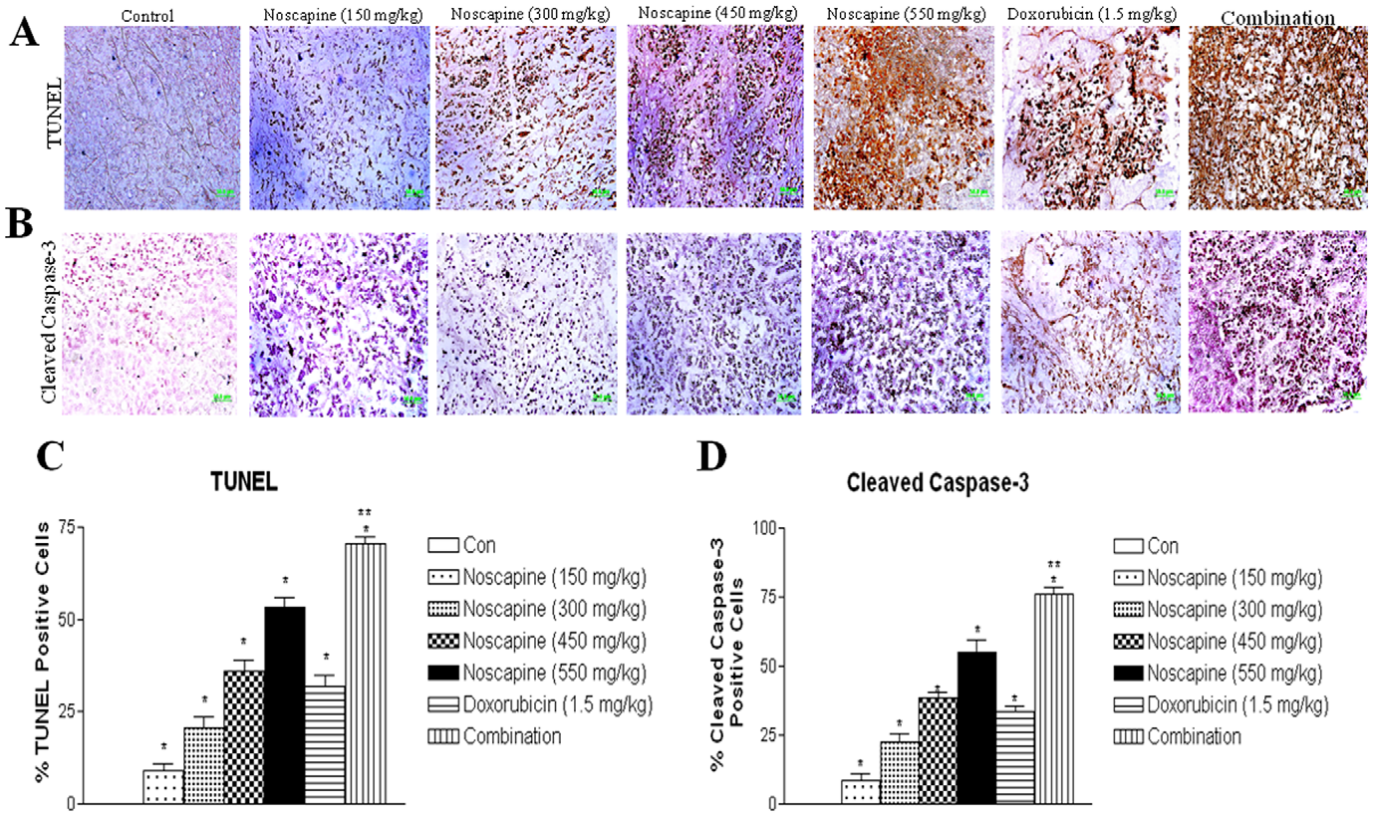
Immunohistochemical staining of xenograft MDA-MB-231 breast tumor tissues for induction of apoptosis using TUNEL assay (A); for expression of cleaved caspase 3 (B); quantitation of apoptotic cells from TUNEL staining (C); and quantitation of caspase 3 positive cells apoptotic cells (D). Tumor sections were stained using the DeadEnd colorimetric kit and cleaved caspase-3 (Asp 175) IHC kit for TUNEL assay and cleaved caspase 3 expression as described in materials and methods respectively. The apoptotic tumor cells are stained brown. Percentages of TUNEL-positive and cleaved caspase 3-positive cells were quantitated by counting 100 cells from 6 random microscopic fields. Data are expressed as mean+SD (N = 6). One-way ANOVA followed by post Tukey test was used for statistical analysis to compare control and treated groups. *P*<0.01 (*, significantly different from untreated controls; ^**^, significantly different from Noscapine and Doxorubicin single treatments). Original magnification ×40 (Micron bar = 100 µm).

To further investigate the role of apoptosis, tumor sections were stained with TUNEL for detection of DNA and expression of cleaved caspase-3 (Fig. 5). Noscapine (150–550 mg/kg/day) treated regressed tumors showed DNA fragmentation (Fig. 5A and 5C) and widespread staining of activated cleaved caspase-3 expression compared to controls (Fig. 5B and 5D), indicating that Noscapine induced apoptosis in a dose dependent fashion of MDA-MB-231 breast cancer cells *in-vivo*. Single-agent treatment with either Noscapine or Doxorubicin induced DNA fragmentation (brown staining) that was further significantly (** *P*<0.001) increased by combination treatment. The combination treatment led to apoptosis in 65±5 percent of the tumor cells, whereas Noscapine and Doxorubicin induced apoptosis in percent 20±3 and 32±3 percent of the tumor cells respectively (Fig. 5A and 5C). Doxorubicin, Noscapine, and combination induced caspase-3 expression in tumors which was significantly (*P*<0.01) different compared to control tumors (Fig. 5 B and 5D). Combination, Doxorubicin and Noscapine treatment showed 68±4, 33±2, and 22±3 percent increased expression of cleaved caspase 3 in tumors tissues respectively compared to control group (Fig. 5B and 5D).

### Inhibition of angiogenesis by combination in MDA-MB-231 tumors

**Figure 6:**
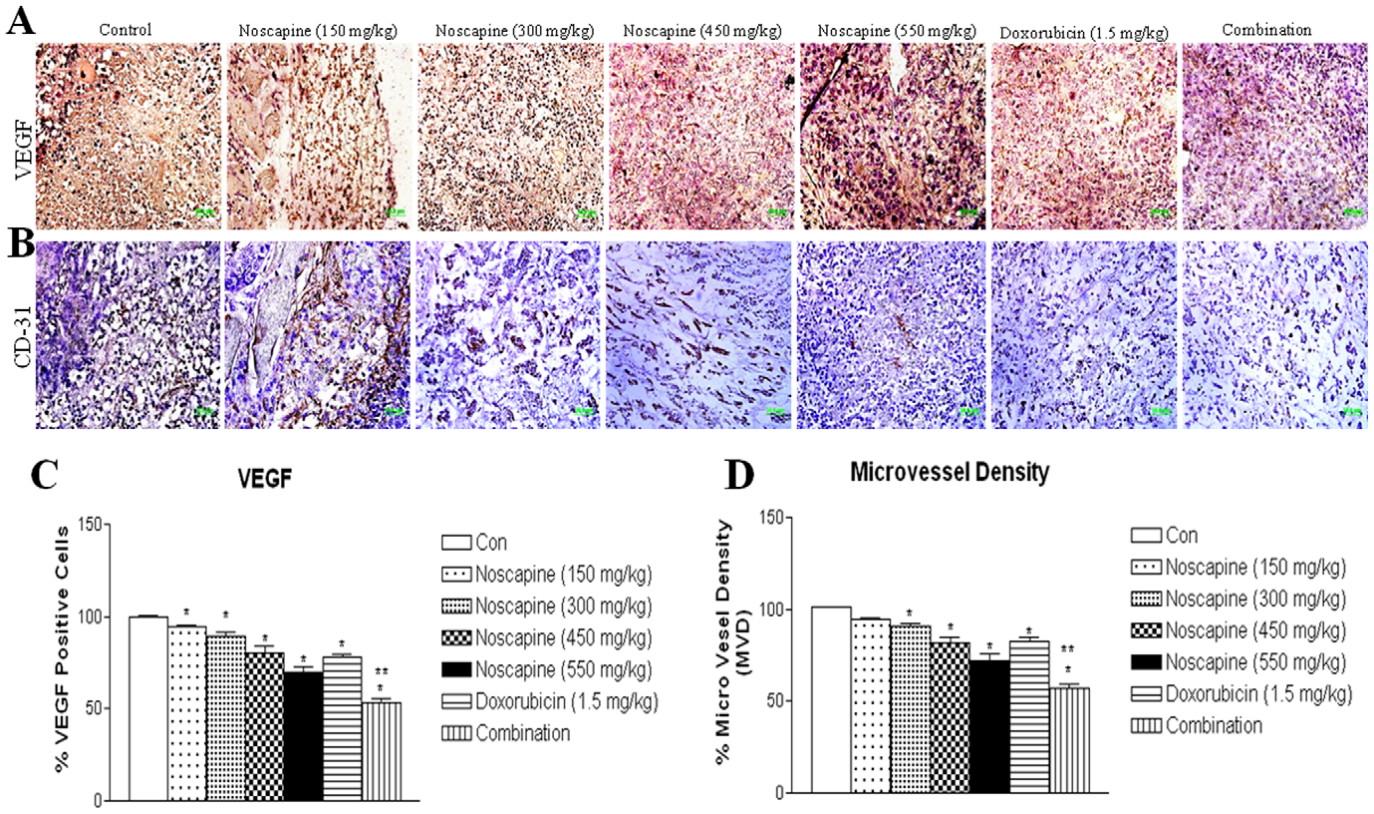
Immunohistochemical staining of MDA-MB-231 tumor tissues for (A) VEGF expression. Tumor sections were stained using the ABC staining kit as described in Materials and Methods. Cells showing positive VEGF expression are stained brown. Original magnification ×40. (Micron bar = 100 µm). Immunohistochemical staining of MDA-MB-231 tumor tissues for (**B**) CD31 expression. Tumor angiogenesis was assessed by immunohistochemical staining with anti-CD31 antibody (brown) on paraffin-embedded sections. Original magnification ×40. (Micron bar = 100 µm). (**C**) Quantitation of apoptotic cells from VEGF staining. (**D**) Assessment of microvessel density. Microvessel density (MVD) was calculated by selecting three most vascularised areas of the tumour (‘hot spots’) and mean values obtained by counting vessels. A single microvessel was defined as a discrete cluster of cells positive for CD31 staining, with no requirement for the presence of a lumen. Microvessel counts were performed at ×400 (×40 objective lens and ×10 ocular lens; 0.74 mm^2^ per field). The MVD was significantly different between the control group and treated groups in sequential analysis; **, *P*<0.01;* *P*<0.05 relative to control.

The highest expression of VEGF was seen in tumor tissues harvested from untreated mice (Fig. 6A & 6C). Decreased VEGF staining was observed in tumors treated with Noscapine (150–550 mg/kg/day) in dose dependent manner and combination (0.44-fold) compared to tumors treated with Doxorubicin (0.21-fold) or Noscapine 300 mg/kg/day (0.1-fold) alone (Fig. 6A and 6C). CD31^+^ endothelial cells were identified using IHC technique in harvested tumor tissues and the results are shown in Fig. 6B and 6D. The staining of CD31^+^ in Noscapine treated groups were significantly decreased to 0.05, 0.09, 0.18, and 0.28-fold at doses of 150, 300, 450, and 550 mg/kg/day compared to control group respectively. The staining of CD31^+^ in combination, Doxorubicin (1.5 mg/kg) and Noscapine (300 mg/kg) treated groups were significantly decreased to 0.40, 0.17, and 0.09-fold compared to control group. The average microvessel per field in groups treated with Noscapine, Doxorubicin and combination were found to decreased by 10±2.6 (*P*<0.05), 17.6±3.5 (*P*<0.01), and 40.6±5.0 (*P*<0.001) respectively compared control group.

## Discussion

TNBC has a more aggressive clinical course than other forms of breast cancer [1], [4]. Traditionally, chemotherapy has been the mainstay of systemic treatment for TNBC since currently available endocrine and HER2-directed therapies are ineffective. [21] Among anticancer agents, antimicrotubules (taxanes and vinca alkaloids) constitute one of the most effective chemotherapeutic agents for treatment of breast cancers. [7] However, their clinical utility is limited due to the development of drug resistance and associated severe side effects. [9], [10] Noscapine is a safer orally active antimicrotubule agent showed in-vitro and in-vivo antitumor activity against variety of cancers including tumors resistant to conventional antimicrotubular agents [13], [14], [15], [16], [17], [18], [19] and did not exhibit severe side effects that are commonly seen with many chemotherapeutic agents. [13], [14] Doxorubicin is an anthracycline drug which has shown significant anticancer activity against TNBC; [22] however its clinical utility has been limited due to low therapeutic index and associated adverse side effects [23]. In the present study, we demonstrated that Noscapine in combination with Doxorubicin was effective in a synergistic manner in inhibition of tumor growth of TNBC both in vitro and in vivo. Our results indicate that the anticancer activity of Noscapine alone and in combination with Doxorubicin was mediated via inactivation of NF-KB, induction of apoptosis, and inhibition of angiogenesis.

To our knowledge, this is the first study that demonstrates effectiveness of Noscapine in combination with Dox against TNBC. In this study, we demonstrated that Noscapine inhibits MDA-MB-231 and MDA-MB-468 cells proliferation *in-vitro* with IC_50_ value of 36.16±3.76 and 42.7±4.3 µM respectively which was comparable with IC_50_ observed with MCF-7 breast (IC_50_ = 42.3 µM), HeLa (IC_50_ = 25 µM), and thymocyte (IC_50_ = 10 µM) cells. [19] The antiproliferative activity of Noscapine was found to vary with the type and sensitivity of cancer cells. We used MDA-MB-231 and MDA-MB-468 TNBC cells to ascertain interaction between Noscapine (sub IC_50_ concentration) and Doxorubicin using isobolographic method. We selected the isobolographic analysis method since it has been widely used to evaluate the interaction between two antitumor drugs and provide both qualitative and quantitative measure of nature and extent of drug interaction. [26] In the present investigation, isobolographic analysis showed that Noscapine enhanced the cytotoxicity of Doxorubicin (CI values<0.59) in MDA-MB-231 and MDA-MB-468 cells in a synergistic manner (Table 1). We recently reported that the CI values of <1.0 are indicative of synergistic interactions in A549 and H460 cells using DIM-C-pPhC_6_H_5_ in combination with Docetaxel [25]. Similarly, our recently conducted studies showed that the interaction between Noscapine and Cisplatin was synergistic (CI<0.6) against non–small cell lung cancer H460 and A549 cells [27]. Also, Hiser et al demonstrated that Noscapine in combination with vincristine showed synergistic (CI<1) in vitro against acute lymphoblastic CCRF-CEM and acute myelogenous leukemia HL-60 cells. [28] Inhibition of cell proliferation and/or induction of apoptotic cell death are key mechanisms by which chemotherapeutic agents exert their action [29]. To study the possible mechanism involved in the anticancer activity of Noscapine and combination, we evaluated induction of apoptosis of MDA-MB-231 cells. The TUNEL assay results showed induction of apoptosis at Noscapine 30 µM alone and in combination with Doxorubicin at 0.4 µg/ml of compared to untreated cells which was evident from positive TUNEL staining and chromatin condensation (Fig. 1). Induction of apoptosis has been demonstrated following Noscapine treatment in various types of human cancer cells like ovarian [14], H460 lung, [18] Hela and mouse thymocytes [19]. Combination treatment showed significant (P<0.001) induction of apoptosis in a synergistic manner in compared to single agent (Fig. 2). Similar to our results, combination treatment of Noscapine (150 mg/kg by gavage once daily) and ^60^Co radiation (single fraction - 25 Gy) showed significant (*P*<0.01) decreased proliferation and increased apoptosis (TUNEL positive cells) of GL261 tumor cells compared to single agent treatment [30].

After establishing the efficacy of Noscapine alone and combination with Doxorubicin on TNBC cells in-vitro, we designed in-vivo experiments to test the efficacy of Noscapine in combination with Doxorubicin against MDA-MB-231 xenograft model in nude mice. Our previous studies have shown dose dependant tumor reduction following oral administration of Noscapine (300–550 mg/kg) against lung cancer [18]. Therefore, we evaluated Noscapine *in-vivo* antitumor efficacy at different dose levels ranging from 150 to 550 mg/kg/day against MDA-MB-231 xenograft model in mice administered by oral gavage. Our results demonstrated that Noscapine at 550 mg/kg (p<0.01) through oral gavage showed higher reduction in tumor volume in MDA-MB-231 xenograft model compared to lower doses used (Fig. 3A). Similarly, Aneja et al reported that oral Noscapine (300 mg/kg) was able to suppress breast cancer progression in a xenograft model (s.c. inoculated 10^6^ MDA-MB-231 cells) by 66 percent compared to control treatment at 24 days post tumor inoculation [20]. We observed 29 percent reduction in the tumor volume following Noscapine 300 mg/kg/day treatment compared to control due to use of 3×10^6^ MDA-MB-231 cells for s.c. inoculation and assessment of in vivo efficacy at 38 days post tumor inoculation.

Previous reports have indicated that Noscapine (120 mg/kg/day, intraperitoneally) was effective in regressing MCF-7 breast tumors but the cell lines used were different than in our study. [19] In another investigation, oral administration of Noscapine at a dose of 300 mg/kg/day showed a significant regression of melanoma tumors compared to untreated animals [13]. The dose dependant antitumor activity of Noscapine in TNBC xenograft model may be attributed to: a) short plasma half life and its availability at the tumor site; and b) extensive first-pass metabolism that reduces the oral bioavailability of Noscapine [31], [32]. There are very few reports available in literature on oral Noscapine absorption and pharmacokinetic parameters and a detailed systematic study is desirable. Our future studies will focus on improving the tumor targeting of Noscapine, so that it is effective at lower doses.

We evaluated the in vivo antitumor efficacy of combination in MDA-MB-231 xenograft tumors in Nu/nu mice using sub therapeutic dose of Noscapine 300 mg/kg/day administered by oral gavage and Doxorubicin 1.5 mg/kg/week by i.v. injection. Our in vivo results demonstrate synergistic behavior of combination in murine MDA-MB-231 xenograft tumor model. Interestingly, Noscapine, Doxorubicin and combination treatment showed non-significant change in weight loss suggesting favorable toxicity profile of Noscapine and Doxorubicin (Fig. 3C). Combination treatment will be advantageous over conventional taxane based combination therapy in treatment of TNBC due to improved patient compliance by oral administration of Noscapine with minimal adverse side effects. Landen et al. showed that in vivo anticancer activity of Noscapine at 300 mg/kg was comparable to that of paclitaxel 25 mg/kg against murine B16LS9 xenograft melanoma model [13]. However, neither additive nor synergistic effect was observed with Noscapine and Paclitaxel combination. Studies in our laboratory also showed that Noscapine and Docetaxel combination was neither additive nor synergistic against NSCLC both in vitro and in vivo which suggests that there was possibly a competition for the same target (unpublished data). On the contrary, our recent investigation demonstrated that Noscapine enhanced the anticancer activity of Cisplatin in an additive to synergistic manner against H460 lung xenografts model [27].

Several studies have provided evidence that enhanced tumor growth inhibition of breast tumors can be achieved by combining Doxorubicin with other agents such as zoledronic acid [33], interleukin-2 [34], TGFβ Inhibitor [35], tetrathiomolybdate [36] as opposed to treating with single agent. *In vivo* studies by Bandyopadhyay et al showed that the small TGFβ Inhibitor, TGFβ1 (1 mg/kg every alternative day) and Doxorubicin (4 mg/kg or 8 mg/kg once per 7 days) combination reduced tumor growth and lung metastasis by inhibition of epithelial-mesenchymal transition in the 4T1 orthotopic xenograft model in comparison to single treatments [35] and these results are consistent with those observed in our study.

To elucidate the underlying mechanism of action of Noscapine and combination treatment, we have evaluated the expression of NF-κB signaling, apoptotic, angiogenic and cell survival proteins using western blot. NF-κB mediates survival signals that inhibit apoptosis as well as promote cancer cell growth. [37] Recent reports indicate that Noscapine exerts inhibitory effect on NF-KB activation [38] and in our studies Noscapine and combination treatment inhibited NF-κB activation through inhibition of IKK activation, IκBα phosphorylation, and IκBα degradation in MDA-MB-231 xenograft TBNC tumors. Previous studies demonstrated that Noscapine induces multiple proapoptotic responses that induce apoptosis against variety of tumors [14], [18], [39], [40], [41], [42]. Caspases are critical protease mediators of apoptosis triggered by different stimuli. [43] In the present study, we found that the Noscapine and combination treatment activated initiator caspases, such as caspase-8 and caspase-9 followed by activation of effector caspase-3. Results of our in vivo studies also demonstrate that Noscapine alone and combination treatment induced proapototic (Bax) or decreased Bcl_2_ proteins (Fig. 3A and 3B) suggesting involvement of mitochondrial pathway. [39] Induction of apoptosis and expression of cleaved caspase 3 was significantly induced in vivo by combination treatment compared to Noscapine or Doxorubicin alone thus confirming that apoptosis is an important pathway associated with the anticancer activity of these compounds (Fig. 5A–D). Our recently published studies with Noscapine and Cisplatin combination also showed that their anticancer activity was mediated by induction of apoptosis via intrinsic and extrinsic pathways and inhibition of survival proteins in H460 lung tumors [27]. Ye et al demonstrated increased apoptotic activity in regressed tumor tissues following Noscapine treatment at 120 mg/kg/day against MCF-7 breast and Renal 1983 bladder tumor xenografts [19]. Our previous studies also showed induction of apoptosis and activation of cleaved caspase 3 following Noscapine treatment in the dose range of 300–550 mg/kg/day in H460 xenografts [18]. Our *in-vivo* results showed increased apoptotic activity and correlated very well with our *in-vitro* results. Wang et al demonstrated that Zoledronic Acid and Doxorubicin combination therapy led to significant (*P*<0.05) increase in caspase-3–positive cells in MDA-G8 breast tumor xenografts compared to single agent therapy. [33] To gain more insights on the anticancer mechanisms of combination therapy, other non-apoptotic signaling pathways need to be investigated and these studies are in progress.

Angiogenesis is critical for establishing solid tumor growth and metastasis [44]. In this investigation, we observed that Noscapine and combination treatment significantly (p<0.001) decreased expression of VEGF (Fig. 4D) in regressed tumors compared to single agent treatment thereby suggesting inhibition of angiogenesis. In addition, Doxorubicin, Noscapine and combination treatment decreased expression of cell survival protein survivin which promotes angiogenesis in TNBC tumors (Fig. 4A and 4B). Survivin was strongly upregulated in angiogenically stimulated endothelium *in vitro* and *in vivo* which protects endothelial cells from apoptosis. [44] The down regulation of survivin was correlated with down regulation of VEGF by Noscapine and combination treatment. Survivin also inhibits caspase activation and acts as a negative regulator [45]. Therefore, the down-regulation of survivin expression results in activation of caspases and thereby induces apoptosis in tumor cells. Furthermore, our IHC results show that Noscapine and combination treatment decreased VEGF staining in tumor tissues harvested from mice compared to single agent treatment and control (Fig. 6A and 6C). The tumor regression by Noscapine and combination was also mediated through decreased expression of VEGF and correlated very well with our VEGF expression results obtained with western blots of tumor lysates (Fig. 4D). MVD is a commonly used index of tumor angiogenic activity and we counted density of neovessels in histological sections of the tumor using CD31 staining. The CD31 expression (Fig. 6 B) and the average microvessels per field (Fig. 6D) in combination treated group were significantly (p<0.001) decreased compared to the single agent treated and control group. The decreased expression of angiogenic markers (IHC) and MVD in treatment compared to control groups appeared to correlate with our western blot results. Thus, Noscapine alone and in combination with Doxorubicin exhibited antiangiogenic activity, and the underlying mechanism is currently being further investigated in our laboratories.

In conclusion, our data provides compelling evidence that combination treatment is effective against MDA-MB-231 TNBC cells as well as tumor xenografts by inactivation of NF-κB, induction of apoptosis and inhibition of angiogenesis. While, the currently available chemotherapeutic agents are associated debilitating toxic side effects, oral Noscapine provides promise as an effective anticancer agent with significantly lower toxicity on normal cells. Thus the use of synergistically acting Noscapine and Doxorubicin combination therapy could be an innovative and promising therapeutic strategy for the treatment for TNBC and possibly will have fewer adverse side effects compared to currently available chemotherapeutic regimens.
